# The Parliamentary Scholar Scheme: a way to engage doctors in healthcare policy and politics

**DOI:** 10.1192/bjb.2019.76

**Published:** 2020-06

**Authors:** Jen Perry, Paul Lomax, Fiona Taylor, Susan Howson, Kathleen McCurdy

**Affiliations:** 1 Camden and Islington Mental Health Trust, UK; 2 South West London and St George's Mental Health NHS Trust, UK; 3 Devon Partnership Trust, Exeter, UK; 4 Oxleas NHS Foundation Trust, Dartford, UK

**Keywords:** Education and training, healthcare policy, politics

## Abstract

The Royal College of Psychiatrists’ Parliamentary Scholar Scheme gives higher trainees in psychiatry the opportunity to spend 1 day a week in the House of Lords working alongside a peer with an interest in health. This article describes the work of the House of Lords and Parliament using examples from the experiences of 2017–2018 scholars and outlines ways doctors can get more involved in policy and politics.

The Royal College of Psychiatrists set up its Parliamentary Scholar Scheme in 2017 with Professor the Baroness Hollins, who is a cross-bench peer in the House of Lords. Professor the Baroness Hollins, a former Consultant Psychiatrist and College President, spends much of her time in the Lords campaigning to improve services for people with learning disabilities, which is one of her specialist areas of interest. The Parliamentary Scholar Scheme, now supported also by the BMA Foundation, gives higher trainees working in psychiatry the opportunity to spend 1 day a week, as a special interest session, in the House of Lords working alongside a peer with an interest in mental health. Peers on the scheme come from across the political spectrum; in the 2017–2018 cohort there were peers from the Conservative, Labour and Liberal Democrat parties as well as from the cross-benches.

The scheme gives trainee psychiatrists hands-on experience of working in the field of health policy as well as wider politics. The psychiatric higher trainee curriculum requires the development of ‘effective leadership skills’ and ‘an understanding of organisational policy and practice at a national and local level in the wider health and social care economy’.^1^ The scheme clearly allows participants to develop such skills but it aims to go further – to enable the trainees to develop skills in influencing the mental health agenda.

There are other schemes available to support trainees to develop leadership skills, for example the National Medical Directors Fellowship Scheme^2^ and the Darzi fellowship scheme.^3^ However, as far as we are aware, the Parliamentary Scholar Scheme is the first programme to give trainees experience of working in the House of Lords. Given the current recruitment and retention problems within the mental health workforce,^4^ this scheme is certainly something that makes psychiatry training stand out from other specialties and may help encourage more doctors into the profession.

This article aims to guide readers through the work of Parliament, mainly focusing on the House of Lords, using examples from our experiences as 2017–2018 scholars.

## What the House of Lords and Parliament do

The UK Parliament is made up of the House of Commons, House of Lords and the monarch. Members of the House of Lords, known as peers, are appointed by the Queen (the monarch) on the advice of the Prime Minister and are unelected. The House of Lords has three main roles: making laws, debating public policy and holding government to account. Members of Parliament (MPs) have the power to overrule the Lords, but the Lords can still be very influential in terms of making/changing legislation and policy.

## Members of the House of Lords

Peers are often experts with experience from outside of Parliament, for example, the peers we worked with had experience of working in psychiatry, nursing, surgery, trade unions and the civil service. Most are ‘life peers’, although 92 (at the time of writing) sit by virtue of a hereditary title. Life peers are appointed by the monarch on the advice of the Prime Minister to serve for life; the title is not transferable when they die.^5^ Peers are nominated either by their political parties or by the House of Lords Appointments Commission, which recommends people for appointment as non party-political life peers.^6^

## Work of the House of Lords

### Making and changing laws (legislation)

Bills (draft laws) are introduced to Parliament and then repeatedly reviewed, debated and amended by both the Lords and the Commons. There is a process known as ‘ping-pong’ where the Bill goes back and forth between each House as amendments are made and agreed or rejected. When the Bill is agreed by both Houses it is given Royal Assent and becomes an Act of Parliament.^8^ An example of a Bill that was going through Parliament while the 2017–2018 scholars were in post was the Mental Health Units (Use of Force) Bill, otherwise known as ‘Seni's Law’. The Bill was proposed by Steve Reed, MP for Croydon North, following the death of one of his constituents, Seni Lewis, in 2010. Seni died aged 23, after being restrained on a mental health ward by 11 police officers. The Bill started in the Commons, went to the Lords, amendments were considered in both Houses and then it became an Act in 2018. The new legislation means that mental health units will have to take steps, including better training for staff, to reduce the use of force against patients. Robust data on the use of force will be collected and police will have to wear body cameras when called to mental health settings; recordings from these cameras can be used in evidence.^9,10^

Another example is the Mental Capacity (Amendment) Bill, which is now enacted^11^ and lays out a new legal framework to replace Deprivation of Liberty Safeguards. Some of the 2017–2018 scholars were able to research the framework, liaise with experts and organisations with interests in the area, and contribute to topics such as the role of care home managers in overseeing the safeguards and the central importance of a person's wishes and feelings when decisions are made about them. Many peers spoke on this Bill as it passed through the House of Lords and some of the 2017–2018 scholars were able to support their peers in drafting speeches using their own experience of working in mental health services and research briefings.

One peer put down an amendment to the EU Withdrawal Bill when it passed through the House of Lords in 2018. The amendment focused on the mutual recognition of professional qualifications, which is about ensuring that professional qualifications (e.g. medical degrees) continue to be recognised in the EU and UK after Brexit. The 2017–2018 scholar attached to this peer undertook some research in this area using a briefing from the Parliamentary library and liaising with relevant organisations, such as the British Medical Association (BMA) and legal firms to seek their perspectives. The scholar then used this information to draft a speech for their peer for the debate on the Bill.^12^

### Debating public policy

Alongside debates on specific legislation there are also debates on topical issues and public policy. It is during these that members are able to give speeches, giving their opinions and arguments and the relevant government minister has to respond. Members may speak because they have a particular interest in the area of debate or particular expertise. The 2017–2018 scholars had the opportunity to contribute to a variety of speeches for their peers on topics related to mental health, for example for debates on access to mental health services for people from Black and minority ethnic groups^13^ and to debates on wider healthcare system issues such as long-term NHS sustainability and global nursing.^14,15^

### Checking and challenging the work of the government (scrutiny)

#### Select committees

MPs and peers hold the government to account. One way of doing this is through the select committees run in the Commons and the Lords. The most important one for health is the House of Commons Health and Social Care Select Committee, which conducts inquiries on a range of topics. Anyone can submit a proposal to a select committee and, as a group of scholars, we submitted a proposal for an inquiry into the state of drug and alcohol services in England. An inquiry we followed during our time in Parliament focused on the impact of Brexit on medicines, medical devices and substances of human origin. Experts (including doctors), interested organisations and members of the public can submit written evidence to inquiries, for example in this one, the BMA and the Academy of Medical Royal Colleges both submitted evidence. The committee also took oral evidence from a range of expert witnesses, including the Rt Hon Jeremy Hunt (the then Secretary of State for Health and Social Care) and Dr Ian Hudson (Chief Executive, Medicines and Healthcare products Regulatory Agency), which some of the 2017–2018 scholars were able to watch. The committee used this evidence to write a report with a series of recommendations. For example, one of the recommendations was that the government should ‘produce a comprehensive list of all the issues relating to the supply of medicines, medical devices and substances of human origin which require contingency planning for the UK leaving the EU […] with evidence that plans are in place to address identified risks to patients’.^17^ The government has to respond to each published select committee report and to consider its recommendations, which may or may not influence government policy. The government published its response to this Brexit report in July 2018 and in answer to the recommendation above it said, ‘At this stage we do not have plans to publish a comprehensive list of the issues relating to medicines, medical devices and substances of human origin. We will continue to be as transparent as possible, but whilst we are engaged in on-going negotiations it is vitally important that we manage information carefully in order to not disadvantage the UK's position’.^18^

#### Written and oral questions

Members also hold the government to account by asking oral or written questions that the government is required to formally answer on the record. Questions on health and social care are answered by the Ministers for Health and Social Care. At the time of writing, Matt Hancock MP is Secretary of State for Health and Social Care. However, there are other government health ministers to be aware of, for example Jackie Doyle Price MP is currently the Parliamentary Under Secretary of State for Mental Health, Inequalities and Suicide Prevention and, in the Lords, the Parliamentary Under Secretary of State (Lords) for Health is Baroness Blackwood. As part of their role, the 2017–2018 scholars drafted oral and written questions that could be used by their peers to put to House of Lords ministers. Ideas for questions came from recently published reports, government announcements and stories in the media.

Oral questions are posed each day in both Houses. In the House of Lords, there is a 30 min slot for four oral questions, which peers have to submit in advance. The peer stands up for their slot and puts their question to the minister for the appropriate department, who has to respond; there is then time for other peers to ask the minister supplementary questions on that topic. The 2017–2018 scholars also identified upcoming oral questions in the chamber that could be of interest to their peers and drafted supplementary questions to be used in the brief debate to further clarify or challenge the government's position. An example of an oral social care question asked by one of the peers we were working for during our time on the scheme was ‘To ask her Majesty's Government what steps they are taking to support (a) the care sector, and (b) those receiving care, in the light of the retrospective change in guidance on the application of the national minimum wage to sleep-in shifts for care workers’.^19^ This question was asked following a widely publicised media story about sleep-in carers being able to claim minimum wage for overnight shifts and was answered by Lord O'Shaughnessy, the then Parliamentary Under Secretary of State (Lords) for Health.

Peers and MPs can submit written questions to government departments that ministers have to respond to within certain time frames. Peers can table up to six questions each day and can expect an answer within 14 days. For example, when the report by the Parliamentary and Health Services Ombudsman on NHS eating disorder services^20^ was published, one of the shadowed peers asked a series of questions about improvements that could be made to medical training and funding, one of which was, ‘To ask Her Majesty's Government, following the conclusions of the Parliamentary and Health Services Ombudsman, *Ignoring the alarms: how NHS eating disorder services are failing patients* (HC 634), published on 6 December, what assessment they have made of the recommendations set out in that report; and what discussions they have held with the General Medical Council on reviewing the eating disorders training for junior doctors’. This was answered by Lord O'Shaughnessy.^21^

### Other work of peers

All-party parliamentary groups (APPGs) are informal cross-party groups that have no official status within Parliament. They are run by and for members of the Commons and Lords. Many choose to involve individuals and organisations from outside Parliament in their administration and activities. Examples are the Acquired Brain Injury APPG, the Mental Health APPG and the Psychology APPG.^22^ The 2017–2018 scholars were able to attend meetings and contribute to the work of some of the APPGs. In 2017–2018 the APPG for Mental Health was chaired by Helen Whateley MP and its secretariat was provided by the Royal College of Psychiatrists and Rethink. Some of the 2017–2018 scholars had the opportunity to work on the APPG for Mental Health's inquiry into the Five Year Forward View for Mental Health.^23^ One of the scholars went on a visit to see some of the new services set by Central and North West London NHS Foundation Trust as a result of the Five Year Forward View and to understand the challenges and opportunities involved. We helped with reviewing evidence and recommendations for the report using our clinical expertise.

Day to day, most peers have meetings with a wide range of people, such as politicians, representatives from charities, think-tanks and NHS organisations, journalists and lobbyists. The 2017–2018 scholars had the opportunity to shadow peers and also contribute to some of these meetings. There are always events taking place in Westminster, for example we were able to accompany our peers to events such as the launch of the report by the Lancet Commission on Liver Disease, the launch of the Schizophrenia Commission report and the Parliamentary Conference on Mindfulness.

Peers also receive correspondence from a wide variety of sources, including members of the public, interested organisations and other politicians. The 2017–2018 scholars were able to help their peers with responding to enquiries and drafting letters.

## The role of the College

Our links with the Royal College of Psychiatrists were invaluable, and the advice from its Public Affairs team enabled us to navigate what can be a complex parliamentary process. They were also able to support us with our parliamentary research on specific topics related to mental health. More broadly, the Public Affairs team works with parliamentarians, arm's-length bodies and other political stakeholders to campaign and influence mental healthcare. It sends out written briefings to parliamentarians on mental health topics which are coming up in debates, oral questions or Bills to give an overview of the topic but also to give the College's perspective (for example with the Mental Capacity Bill). Team members regularly meet with politicians face to face about different mental health issues. The Public Affairs team, alongside Rethink, coordinates the APPG on Mental Health and helps to plan their activities and inquiries for the year. The team also sends out a weekly email to College members entitled ‘Political Week’, which gives an overview of any mental health topics that have come up in Parliament.

## What we learnt and skills we developed

Our 1-year scholarship was an exciting and unique opportunity to learn more about the interface between politics and healthcare and how Parliament works. As trainees it gave us a better understanding of the wider mental health system and its interactions with government. We developed skills in leadership, policy analysis, speech writing and influencing the mental health agenda, all of which will be helpful for us as consultant psychiatrists. Within the Healthcare Leadership Model,^24^ these skills correspond to the ‘connecting our service’ domain, as we were able to develop an understanding of how different services connect to the broader health landscape, how complex relationships form and how decisions are made. It also corresponds to the ‘influencing for results’ domain, as we were able to develop our communication skills and our ability to influence people.

During our time, we were able to meet with a number of MPs and peers who are influential in healthcare to learn more about how they got into politics, their day-to-day work and what their priorities are. In return, we were able to share with them our experiences of working in front-line mental health services. Some of us took our peers to visit our clinical teams so that they could get an in-depth understanding of what it is like to work in psychiatry.

All of us have been able to share our learning with colleagues through teaching sessions, blogs and conference presentations. At present there are no objective data to examine the impact of the scheme, but this could be gathered after further cohorts of scholars have completed the placement.

## How doctors can get involved

There are lots of different ways doctors can get involved in healthcare policy and politics. One way is to join a political party, which will allow you to develop an understanding of the political system and to become politically active. You can write to your local MP, or a peer in the House of Lords with an interest in your issue. Politicians' interests are listed on their Parliament webpage. The select committees regularly run inquiries and, as a doctor, you can submit evidence, propose a topic or go to watch the evidence sessions. You can follow what goes on in the Houses of Commons and Lords by reading Hansard (https://hansard.parliament.uk), watching Parliament TV (https://www.parliamentlive.tv/Commons) or listening to the BBC Radio 4 programme ‘Today in Parliament’ (https://www.bbc.co.uk/programmes/b006qtqd), which provides a 30 min summary of the day's events.

If you have an interest in a particular topic you can campaign for it on social media, write blogs or articles for newspapers or go on TV or radio. The difficulties with the revised NHS junior doctor contract a few years back led to many more doctors becoming politically active. There are also roles for doctors in organisations that lobby government, for example the BMA. Many medical Royal Colleges have a policy and/or parliamentary committee which will influence policy and you may be able to get involved with this. The Royal College of Psychiatrists produces a weekly political update that you can sign up for. This will keep you up to date with forthcoming parliamentary business.

Of course, if you are a psychiatry higher trainee you can apply to the Parliamentary Scholar Scheme, which (at the time of writing) is in its third year. It is advertised on the Royal College of Psychiatrists website usually in the spring.

## Conclusions

Our participation in the 2017–2018 Parliamentary Scholar Scheme was a unique opportunity for us as trainee psychiatrists to learn more about healthcare policy and Parliament. It has enabled us to develop skills in leadership and influencing that will stand us in good stead for our future careers as consultant psychiatrists.

The idea of the medical profession turning to soft power to influence policy has been proposed recently.^25^ Our experience of working in Parliament has demonstrated the many areas where policy is made, challenged and communicated. The scope for those with first-hand knowledge of the healthcare system to have input into the areas where policy is influenced is there, but it requires knowledge of the system and a willingness to suggest solutions, not just to criticise the end product.

## About the authors

**Jen Perry** is a consultant psychiatrist with Camden and Islington Mental Health Trust, London, UK; **Paul Lomax** is a specialty trainee with South West London and St George's Mental Health NHS Trust, UK; **Fiona Taylor** is a consultant psychiatrist with Camden and Islington Mental Health Trust, UK; **Susan Howson** is a consultant psychiatrist with Devon Partnership Trust, Exeter, UK; **Kathleen McCurdy** is a consultant forensic psychiatrist with Oxleas NHS Foundation Trust, Dartford, UK. All five authors were higher trainees on the 2017–2018 Parliamentary Scholar Scheme.
